# Supplementary material for: A Comprehensive Assessment of Ultraviolet-Radiation-Induced Mutations in Flammulina filiformis Using Whole-Genome Resequencing
Source: J Fungi (Basel). 2024 Mar 20;10(3):228. doi: 10.3390/jof10030228 (PMC10971301; doi:10.3390/jof10030228)
Supplement: Supplementary file 1 [file jof-10-00228-s001.zip › Supplementary Material S8/KEGG annotation/out/64381550635650.os/KO/out_map/map04933.html]

KEGG PATHWAY: AGE-RAGE signaling pathway in diabetic complications - Reference pathway


|  |  |
| --- | --- |
| **AGE-RAGE signaling pathway in diabetic complications - Reference pathway** |  |

[
Pathway menu
| Organism menu
| Pathway entry
| Show description
| User data mapping
]

Advanced glycation end products (AGEs) are a complex group of compounds produced through the non-enzymatic glycation and oxidation of proteins, lipids and nucleic acids, primarily due to aging and under certain pathologic condition such as huperglycemia. Some of the best chemically characterized AGEs include N-epsilon-carboxy-methyl-lysine (CML), N-epsilon-carboxy-ethyl-lysine (CEL), and Imidazolone. The major receptor for AGEs, known as receptor for advanced glycation end products (RAGE or AGER), belongs to the immunoglobulin superfamily and has been described as a pattern recognition receptor. AGE/RAGE signaling elicits activation of multiple intracellular signal pathways involving NADPH oxidase, protein kinase C, and MAPKs, then resulting in NF-kappaB activity. NF-kappa B promotes the expression of pro-inflammatory cytokines such as IL-1, IL-6 and TNF-alpha and a variety of atherosclerosis-related genes, including VCAM-1, tissue factor, VEGF, and RAGE. In addition, JAK-STAT-mediated and PI3K-Akt-dependent pathways are induced via RAGE, which in turn participate in cell proliferation and apoptosis respectively. Hypoxia-mediated induction of Egr-1 was also shown to require the AGE-RAGE interaction. The results of these signal transductions have been reported to be the possible mechanism that initates diabetic complications.

|  |  |
| --- | --- |
| Reference pathway | 100% |
